# Author Correction: Camphor-Engine-Driven Micro-Boat Guides Evolution of Chemical Gardens

**DOI:** 10.1038/s41598-019-39974-w

**Published:** 2019-03-12

**Authors:** Mark Frenkel, Victor Multanen, Roman Grynyov, Albina Musin, Yelena Bormashenko, Edward Bormashenko

**Affiliations:** 1 Physics Department, Ariel University, Natural Sciences Faculty, P.O.B. 3, 407000 Ariel, Israel; 2 Department of Chemical Engineering, Biotechnology and Materials, Ariel University, Faculty of Engineering, P.O.B. 3, 407000 Ariel, Israel

Correction to: *Scientific Reports* 10.1038/s41598-017-04337-w, published online 21 June 2017

The Acknowledgements section in this Article is incomplete.

“The authors are indebted to Professor Gene Whyman for fruitful discussions.”

should read:

“The authors are indebted to Professor Gene Whyman for fruitful discussions. Acknowledgement is made to the donors of the Israel Ministry of Absorption for the partial support of the scientific activity of Dr. Mark Frenkel.”

