# Supplementary material for: Camphor-Engine-Driven Micro-Boat Guides Evolution of Chemical Gardens
Source: Sci Rep. 2017 Jun 21;7:3930. doi: 10.1038/s41598-017-04337-w (PMC5479842; doi:10.1038/s41598-017-04337-w)
Supplement: Supplementary file 2 — Supplementary Information [file 41598_2017_4337_MOESM2_ESM.pdf]

Camphor-Engine-Driven Micro-Boat Guides Evolution of Chemical Gardens

Mark Frenkel[a,b], Victor Multanen[b] , Roman Grynyov[a], Albina Musin[a], Yelena Bormashenko[b], Edward Bormashenko,[b*]

[a]*Ariel University, Natural Science Faculty, Physics Department, P.O.B. 3, 407000, Ariel, Israel*

[b]*Ariel University, Engineering Faculty, Chemical Engineering Department, P.O.B. 3, 407000, Ariel, Israel*

[*]Corresponding author: Edward Bormashenko

Ariel University, Natural Science Faculty, Physics Department, Chemical Engineering and Biotechnology Department, P.O.B. 3, Ariel 40700, Israel

Phone: +972-3-906-6134; Fax: +972-3-906-6621

E-mail: edward@ariel.ac.il

Supplementary Video: Heliciform precipitates formed under the self-propulsion of the micro-boat.

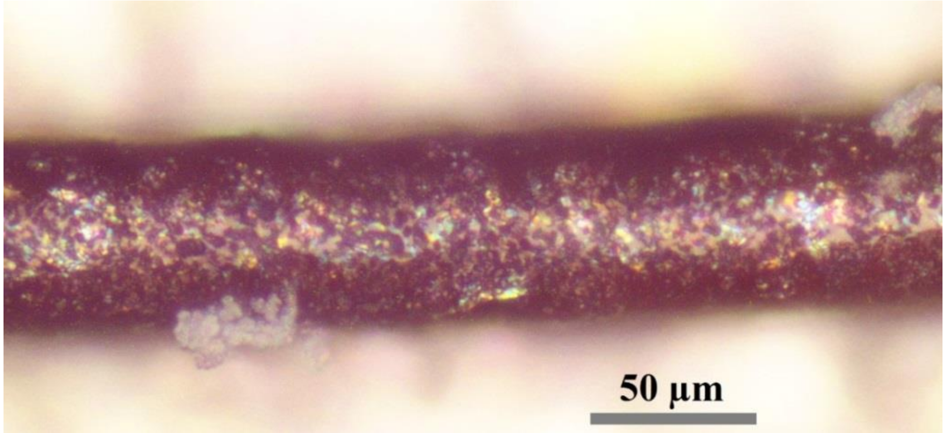

a.

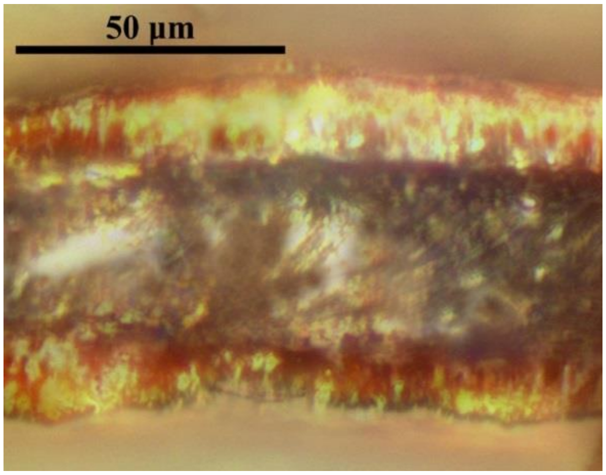

b.

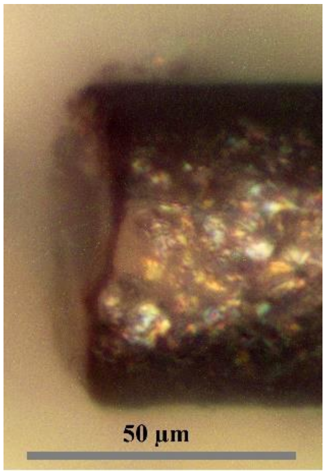

c.

Supplementary Figure S1. a - The filament typical for chemical gardens is shown; the magnification is ×600; b - The same filament cut lengthwise; c - The butt of the filament; the magnification is ×750.

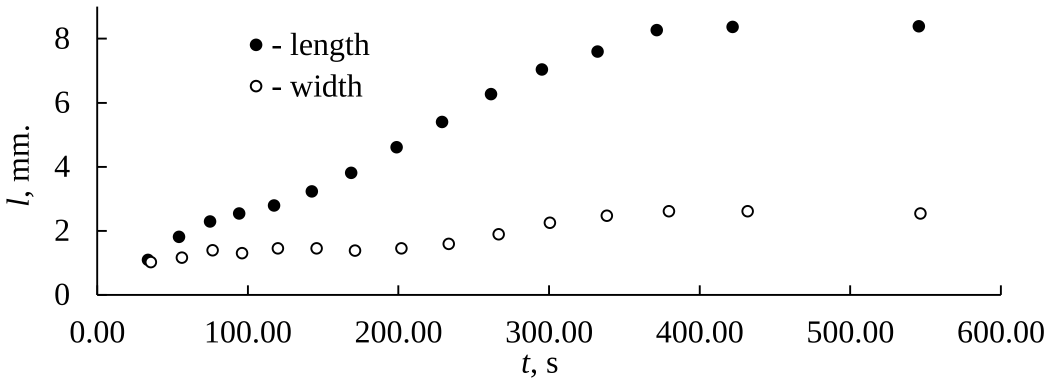

Supplementary Figure S2. The time dependence of changes in geometrical parameters of the precipitate (the concentration of $K_4Fe(CN)_6$ c=2 wt.%).

Supplementary Table S1. Values of fitting parameters $\tilde{a}, \alpha, \tau_d$ and τ_{fr} extracted from the experimental data.

Potassium hexacyanoferrate concentration, c wt.%	Boat length, L, mm	$\tilde{a}$, m/s^2	α	τ_d , s	τ_{fr} , s
2.0 (Fig. 7 a)	15	0.023	1.33×10^{-4}	186.0	3.85
0.125 (Fig. 7 b)	15	0.023	1.3×10^{-4}	31.5	0.73
2.0 (Fig. 7 c)	10	0.021	7.94×10^{-5}	35.3	0.67
0.25 (Fig. 7 c)	15	0.014	8.04×10^{-5}	50.2	0.93

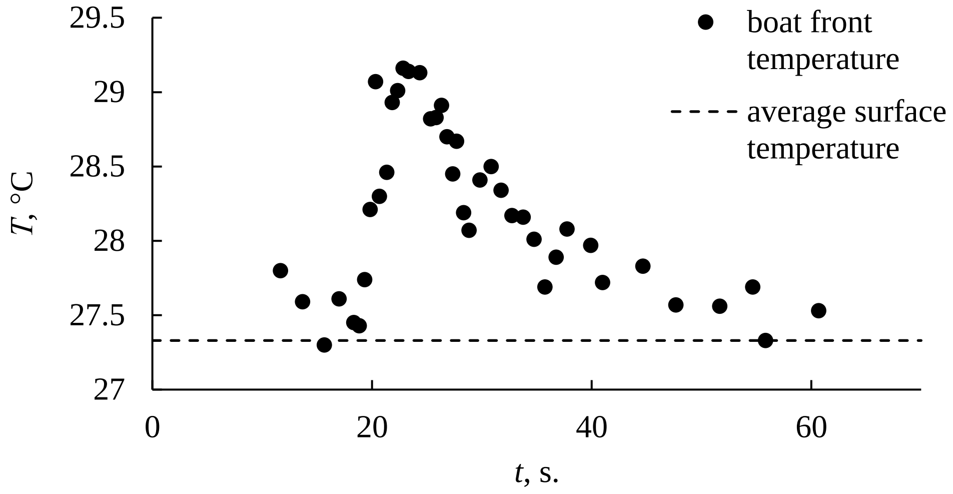

a.

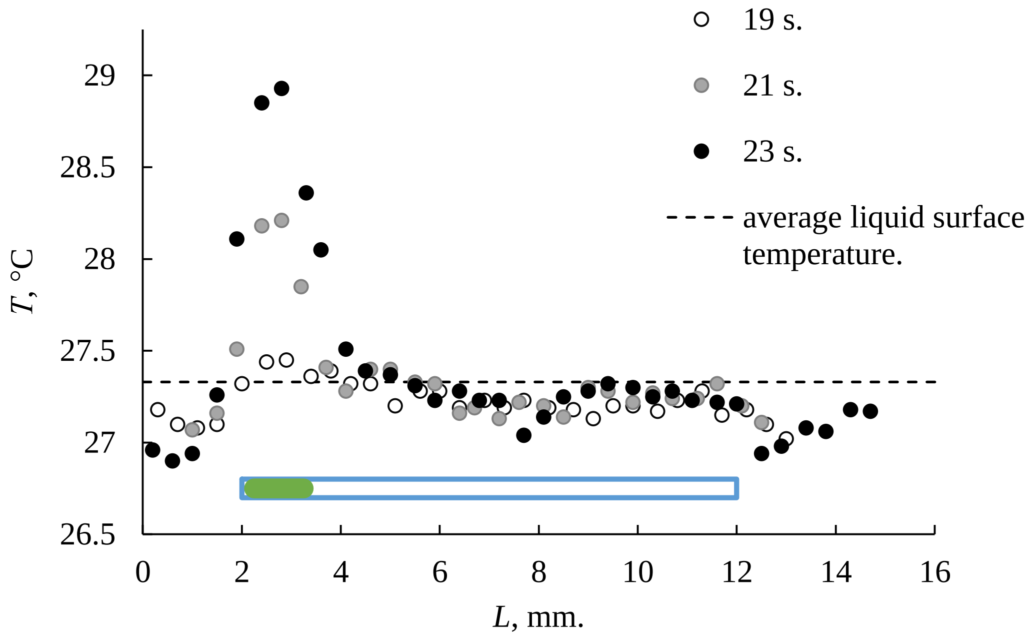

b.

Supplementary Figure S3. **Temporal (a) and spatial (b) temperature distributions during the self-propulsion.** **a** - The time dependence of the temperature of the boat front (*c*=0.25 wt.%). The temperature jump in the initial period of self-locomotion is clearly seen. **b** - The temperature distribution along the micro-boat surface (*c*=0.250 wt.%). The blue rectangle represents the micro-boat in its real dimensions.

The rotational motion of the micro-boat along the rim of the Petri dish should be addressed within the model developed in Ref. 51. Let the vertical axis *Z* be coinciding with the inner side of the rim of the Petri dish, as shown in Supplementary Figure S4.

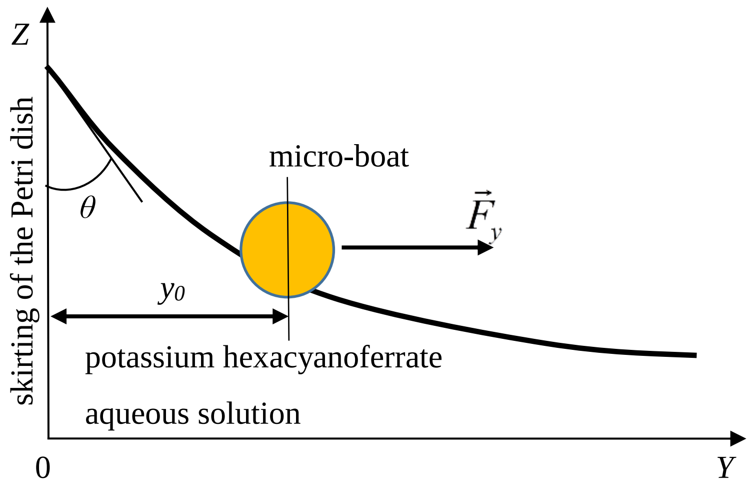

Supplementary Figure S4. The cross-section of the PVC tubing (micro-boat) driven by the camphor engine is depicted with yellow color. The contact angle θ formed by the potassium hexacyanoferrate aqueous solution and the rim of the Petri dish is shown.

The micro-boat rotates in the Petri dish at the distance of *ca*. 1 cm from the rim ($y_0 \cong 1\,cm$ in Fig 6 and Supplementary Fig. S4). Assume that the meniscus formed by the potassium hexacyanoferrate aqueous solution with the rim has the contact angle θ (established experimentally with the goniometer as θ=35±1°; the contact angle hysteresis[52] is neglected). Bush et al. demonstrated that in this case, the lateral force $\vec{F}_y$ acts on the micro-boat[51] (Fig 6 and Supplementary Fig. S4):

$$\left|\vec{F}_y\right| = -T\cot\theta\exp\left(-\frac{y_0}{l_{ca}}\right), \qquad (6)$$

where T is the modulus of the vertical force exerted by a floating body on a liquid/vapor interface, and $l_{ca} = \sqrt{\gamma/\rho g}$ is the capillary length.[52] In our case, the lateral force $\vec{F}_y$ is the centripetal force acting on the micro-boat; thus we obtain:

$$T\cot\theta\exp\left(-\frac{y_0}{l_{ca}}\right) \cong \frac{mv_{cm}^2}{R-y_0}, \qquad (7)$$

where R is the internal radius of the Petri dish. Assuming for the sake of a very rough estimation: $\theta \cong 35^0;\ y_0 \cong 1cm,\ l_{ca} \cong 0.271cm;\ R = 5cm;\ T \cong \frac{1}{2}mg$ yields:

$$v_{cm} \approx \sqrt{\frac{1}{2}(R-y_0)g\exp\left(-\frac{y_0}{l_{ca}}\right)} \cong 8.5\frac{cm}{s}, \qquad (8)$$

which is an overestimated value (friction is neglected) but reasonable, when compared with the experimentally observed characteristic velocity of rotation of micro-boats.
